# Serious Illness Communication Skills Training for Emergency Physicians and Advanced Practice Providers: A Multi-Method Assessment of the Reach and Effectiveness of the Intervention

**DOI:** 10.21203/rs.3.rs-2561749/v1

**Published:** 2023-02-21

**Authors:** Oluwaseun Adeyemi, Alexander D. Ginsburg, Regina Kaur, Allison Cuthel, Nicole Zhao, Nina Siman, Keith Goldfeld, Lillian Liang Emlet, Charles DiMaggio, Rebecca Yamarik, Jean-Baptiste Bouillon-Minois, Joshua Chodosh, Corita R. Grudzen, The PRIM-ER Investigators

**Affiliations:** NYU Grossman School of Medicine; Mayo Clinic; CHI Saint Joseph Health; NYU Grossman School of Medicine; Stony Brook University; NYU Grossman School of Medicine; NYU Grossman School of Medicine; University of Pittsburgh Medical Center; NYU Grossman School of Medicine; VA Long Beach Healthcare System; CHU Clermont-Ferrand; NYU Grossman School of Medicine; Memorial Sloan Kettering Cancer Center; The Ohio State University Wexner Medical Center

**Keywords:** Palliative Care, Emergency medicine, Communication, Education and training

## Abstract

**Background:**

EM Talk is a communication skills training program designed to improve emergency providers’ serious illness conversational skills. Using the Reach, Effectiveness, Adoption, Implementation, and Maintenance (RE-AIM) framework, this study aims to assess the reach of EM Talk and its effectiveness.

**Methods:**

EM Talk is one of the components of Primary Palliative Care for Emergency Medicine (EM) intervention. It consisted of one 4-hour training session during which professional actors used role-plays and active learning to train providers to deliver serious/bad news, express empathy, explore patients’ goals, and formulate care plans. After the training, emergency providers filled out an optional post-intervention survey, which included course reflections. Using a multi-method analytical approach, we analyzed the reach of the intervention quantitatively and the effectiveness of the intervention qualitatively using conceptual content analysis of open-ended responses.

**Results:**

A total of 879 out of 1,029 (85%) EM providers across 33 emergency departments completed the EM Talk training, with the training rate ranging from 63–100%. From the 326 reflections, we identified meaning units across the thematic domains of improved knowledge, attitude, and practice. The main subthemes across the three domains were the acquisition of discussion tips and tricks, improved attitude toward engaging qualifying patients in serious illness (SI) conversations, and commitment to using these learned skills in clinical practice.

**Conclusion:**

Effectively engaging qualifying patients in serious illness conversations requires appropriate communication skills. EM Talk has the potential to improve emergency providers’ knowledge, attitude, and practice of SI communication skills.

**Trial registration:**

NCT03424109

## Introduction

More than half of seriously ill older adults visit the Emergency Department (ED) in the last six months of life.^1,2^ It is estimated that between 50 and 60 percent of seriously ill older adults do not have advanced directives^3,4^ and are at risk of receiving care inconsistent with their wishes.^5^ The ED presents an opportunity to engage these patients in discussions focused on goals of care, advanced directives, and willingness to obtain hospice and palliative care. Initiating serious illness (SI) conversations are never an easy task for providers, irrespective of the specialty.^6–8^ Emergency Medicine (EM) providers particularly tend to avoid such conversations as they are more likely to assume that they are better suited to provide life-prolonging interventions and providers of other specialties are better equipped to handle such conversations.^9^

Educational interventions aimed at improving physicians’ communication skills in SI conversations gained increasing attention about two decades ago,^10^ with the VitalTalk being one of the most commonly used educational training models.^11,12^ A few specialty-focused adaptations of the VitalTalk had emerged over time such as OncoTal*k* for oncology providers,^13,14^ Geritalk for geriatric providers,^15,16^ and EM Talk for emergency providers.^17^ Integral to the VitalTalk training framework are evidence-based pedagogical techniques such as the use of simulated patients and caregivers, role-playing, and small group learning.^16–18^ Earlier studies have reported that the OncoTalk training was associated with a substantial increase in oncology providers acquiring new skills in delivering bad news and transitioning qualifying patients to palliative care.^18,19^ Additionally, the Geritalk training has been associated with substantial improvement in self-reported preparedness and practice of engaging in SI conversations.^20,21^

Unlike medical specialties with a controlled patient-provider environment like primary care and oncology, navigating SI conversations in the ED environment requires additional skills in engaging patients and caregivers in a fast-paced environment while maintaining patient privacy. Furthermore, EM providers are required to demonstrate competency in interpersonal and communication skills including delivering bad news and resolving conflicts, consistent with the guidelines from the Accreditation Council of Graduate Medical Education.^22^ With EM Talk focused on providing communication skills training as part of the educational intervention of a multi-center National Institute of Health grant,^23^ it is unknown how effective the educational intervention improved the knowledge, attitude, and practice of EM providers.

To evaluate this provider-focused educational intervention, we adopted the Reach, Effectiveness, Adoption, Implementation, and Maintenance (RE-AIM) framework.^24–26^ The two-decade-old RE-AIM framework is a planning and evaluation tool commonly used to assess project implementation across clinical, public health, and health behavior-focused research.^24^ For this study, we focused on assessing the intervention’s reach – defined as the absolute number of persons who participated in the intervention, and the intervention’s effectiveness – defined as the impact of the intervention on individual outcome measures.^24^ Therefore, the aim of this study, is to assess the reach of EM Talk and its effectiveness in improving knowledge, attitude, and practice among EM providers.

## Methods

### Study Design

We employed a multi-method approach to assess the reach and effectiveness of the EM Talk intervention in providing SI communication skills for full-time EM physicians and advanced practice providers (hereafter referred to as EM providers). The advanced practice providers involved in SI communication skills training were those involved in the care of high-acuity patients. Consistent with this multi-method research design,^27^ the reach of the intervention was assessed quantitatively using a cross-sectional study design while the effectiveness was assessed qualitatively using a conceptual content analytical design.^24^ We defined the reach of the EM Talk intervention as (1) the absolute number and proportion of representative EM providers across each participating ED that obtained the SI communication skill training, (2) the estimated number of seriously ill patients potentially reached, and (3) the estimated yearly number of patients each trained provider will reach across each ED. We defined the effectiveness of the EM Talk intervention as the self-reported thematic domains of improved knowledge, attitude, and practice of SI communication skills. The unit of analysis of the quantitative and qualitative studies was at the institutional and individual levels, respectively. This study followed the Standards for Reporting Qualitative Research (SRQR) guideline.^28^

### Study Population

The study population was full-time EM providers across 33 EDs enrolled in the Primary Palliative Care for Emergency Medicine (PRIM-ER) study. The PRIM-ER study is a cluster-randomized pragmatic trial that assesses the impact of EM provider interventions on healthcare utilization and outcomes among seriously ill older adults that visit the ED.^23^ The PRIM-ER intervention consists of (1) education in palliative and end-of-life care EM providers and emergency nurses,(2) communication skill training and simulation workshop for EM providers (using the EM Talk training) and emergency nurses (using the End-of-Life Nursing Education Consortium (ELNEC) training), and (3) the integration of a clinical decision support tool to identify and engage seriously ill older adults in SI conversations. We had reported the reach of the ELNEC intervention and emergency nurses’ perceived barriers and solutions to conducting SI conversations in the ED.^29^ The current study focuses on the reach and effectiveness of EM Talk.

### EM Talk Intervention

EM Talk was a one-day 4-hour SI communication skill training session, delivered both in-person and virtually. Consistent with our cluster-randomized stepped wedge design,^23^ EM Talk training occurred sequentially across 33 EDs for three years (2019 to 2021). Before each training session, an EM Champion – an influential EM provider, was selected to encourage and mobilize EM providers for the training and organize the training logistics in his or her ED. The first half of the session comprised large group lectures and the second half of the session focused on small group practice sessions on skills in delivering bad news and discussing goals of care and reflective exercises. Each session was facilitated by two VitalTalk-trained personnel. Details of the EM Talk course description have been published earlier.^17^ Within a week after the SI communication skill training, EM providers completed a self-administered post-training survey and received a five-unit continuing medical education (CME) credit and a $67 gift card for their time.

### Quantitative Data Analysis

We obtained administrative data from each ED of the PRIM-ER study and the Centers for Medicare & Medicaid Services (CMS). Using the administrative data, we computed the counts of the EM providers that completed the EM Talk training and generated the sum of EM providers in each participating ED. Using the CMS data, we generated the yearly number of seriously ill patients that visit the ED by computing the mean of the number of qualifying seriously ill patients that visited the participating sites between 2018 and 2020. A qualifying seriously ill patient is a patient, 66 years or older, that visited the ED within the study period with a life-limiting illness identified using a GAGNE index (a measure of one-year mortality) greater than 6.^23,30^ We defined the proportion of EM providers trained as the number of EM providers trained divided by the total number of EM providers in the participating EDs (Table 1). We defined the estimated number of seriously ill patients as the yearly average number of qualifying seriously ill patients in each ED multiplied by the proportion of EM Talk-trained providers in the ED. We defined the yearly seriously ill patient and EM provider ratio as the average yearly average of the seriously ill patients reached divided by the number of EM providers trained.

### Qualitative Data Analysis

Consistent with a conceptual content analytical approach, we identified codes that fell into three a priori-defined thematic domains of improved knowledge, attitude, and practice. The knowledge, attitude, and practice (KAP) theory, a commonly used theoretical model to assess behavior change, divides the steps in behavioral change into knowledge acquisition, belief and attitude generation, and practice creation. We selected the KAP theory as the conceptual model to assess the effectiveness of the EM Talk intervention since the intent of the intervention was to equip EM providers with communication skills (knowledge acquisition), create a simulated practice experience (attitude generation) so that they can effectively engage seriously ill patients on discussions around goals of care (practice creation).

Data for the qualitative analysis was from three open-ended questions in the EM Talk post-training survey – designed consistent with the requirement of continuing medical education assessment (Appendix 1).^31^ The questions are as follows: (1) *In the space below, please reflect on your personal experience with this educational intervention*; (2) *What changes will you make to how you identify patients and/or family members who may be ready to discuss goals of care and/or palliative care options?* (3) *What changes will you make to how you counsel patients and/or families about end-of-life care?* Responses to these questions were optional and were prefixed as “A”, “B”, and “C”.

Using each respondent’s sentences as the unit of analysis, the coding team, made up of three coders (two males (OA, AG), one female (RK), all MDs), inductively and deductively identified codes and meaning units after an initial textual immersion.^32^ A codebook was generated after analyzing the responses to the first open-ended question and the codebook was continuously modified throughout the analytical phase (Table 2).^33^ Each coder coded the qualitative data pool independently and final codes were agreed upon through voice voting during coding and debriefing meetings. After an initial round of coding (open coding), the coding team performed focused coding, during which the initial codes were merged and recategorized. Meaning units (exemplary sentence or phrasal codes) were generated from the sentences through the use of in-vivo, structural, and process coding techniques, and their counts were reported in tables.^34^ Subthemes were identified by pooling codes with similar meaning units.^32^

We employed several methods to ensure methodological and interpretive rigor. To ensure credibility, the coding team reported the final codebook created after a series of debriefing and coding meetings.^35^ The open-ended questions that informed the responses provide information on the dependability of the study and the details of the study participants and the source of data provide information on the transferability of our findings.^36^ By reporting the counts of the meaning units of each theme and using quotes from the participants to explain the thematic domain, we ensured the confirmability of the study.^37^

### Human Subject Concern

Ethical approval was obtained from the New York University Grossman School of Medicine Institutional Review Board (ID: i18–00607) and the PRIM-ER study protocol is reported on ClinicalTrials.gov (NCT03424109).^38^

## Results

### Quantitative Results: Reach of Intervention

A total of 879 out of 1029 EM providers (85%) completed the EM Talk training (Table 3). The proportion of EM providers that had the training across the 33 EDs ranged from 63–100%. Between 2018 and 2020, a total of 2,698,198 unique patients, 66 years and older, visited the 33 EDs at least once. Of this population, the number (and proportion) of unique seriously ill patients (GAGNE score > 6) was 57,136 (2.1%). The yearly average of seriously ill patients across the 33 EDs was 19,045. We estimated that 16,389 seriously ill patients would have been reached across all 33 EDs, assuming a 100% practice rate among the trained EM providers. Also, we estimated that every year, one trained EM provider will reach an average of 19 qualifying seriously ill patients and the number will vary from 5 to 115 across the 33 EDs.

### Qualitative Results: Effectiveness Of Intervention

**Figure 1:**
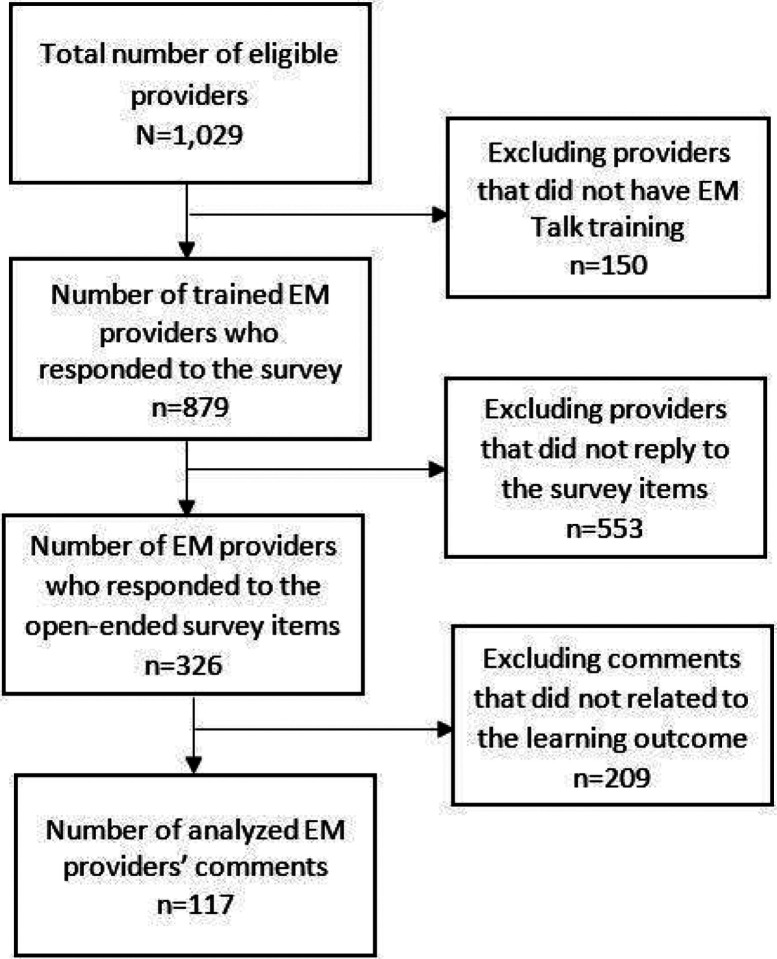
Data selection steps

Across the 879 EM providers who completed the survey, we coded 117 open-ended responses (Fig. 1). Sentences from 60 respondents were coded under the improved knowledge domain while sentences from 45 and 25 respondents were coded under the improved attitude and improved practice domains, respectively. With some sentences producing multiple codes across the thematic domains, the code counts exceeded 117 (Table 4).

### Improved Knowledge

The theme of improved knowledge was referenced by 60 respondents. The most common subthemes that emerged from these responses were the acquisition of tips and tricks of SI communication (n = 44) and acquired general yet useful knowledge (n = 14). A less common subtheme was the development of empathy skills (n = 3) (Table 5).

### Acquired tips and tricks for SI communication

A majority of respondents acknowledged that they “*learned some really valuable tools” (B254)* and that “*the tips and tricks provided were concise and therefore relatively easy to remember with regular practice/use” (B256)*. One provider recounted:

“I did learn some helpful skills that I will try to bring into my practice.”

(A64)

To these trained EM providers, the SI communication skills taught in the course were viewed as “*techniques to talk to the family of palliative patients” (A35)*. One provider highlighted the importance of this skill based on the frequency of contact with SI patients and their caregivers in the EDs:

“This was a useful educational intervention to ED providers who often have to have end-of-life discussions with patients and families in an emergent setting.”

(A34)

### Acquired general useful knowledge

In contrast to EM providers that specified specific skills the EM Talk provided, some providers reported a general improvement in their knowledge of palliative care. For some, the training was *“a pretty good learning experience for me” (C314)* while another provider feels the training *“really helped me grow as a provider” (B194)*. A provider shared:

“I learned more than I thought I would, made me think about these issues more than I had before.”

(A49)

### Acquired empathy skills

The acquisition of empathy skills was the least identified subtheme, with one provider identifying empathy skills as a takeaway from the training.

“Learned a lot about empathetic skills that I can use in daily practice”

(A81)

### Improved Attitude

The theme of improved attitude was present in 46 responses. The most common subtheme that was identified was improved attitudes toward engaging in hospice and palliative care discussions (n = 30). Less frequently identified subthemes included attitude towards improving patient care (n = 10) and attitude towards receiving future training on SI conversations (n = 5) (Table 5).

### Improved attitude toward engaging in SI conversations

The improved attitude towards engaging in SI conversations referred to being “*more comfortable and at ease with end-of-life conversations” (B190)*. For some EM providers, the training helped them “*realize the importance of having discussions with family early/often regarding goals of care for their loved ones” (B188)*.

Some EM providers, however, discussed the deliberate attempt of the EM provider “*to slow down and listen to your patients and family members” (B166)*. The importance of being intentional about listening was stated by one of the EM providers:

“Patients end up being more satisfied when you listen and they feel as if their needs and concerns are being addressed”

(C311)

A few EM providers stated that the training helped increase the motivation to engage in SI conversations. For example, one provider wrote about negative past experiences and how the course made them feel more confident with such conversations:

“…due to time constraints and some negative patient interactions regarding the goal of care discussions, I was initially resistant but now motivated and optimistic in my ability to navigate these talks”.

(A63)

### Improved attitude toward patient care

Improved attitude towards patient care referred to the EM providers “*see(ing) the value [the training] brings to patients and their families” (B208)*. The training provided an opportunity for self-reflection and assessment with one provider stating that “*I identified various areas in which I can improve not only my communication in end-of-life discussions but also with all my patients” (B258)*. The awareness of how the training may improve patient care served as a motivation for some EM providers to practice SI conversations.

“[The course] pushed my comfort level with these discussions and has motivated me to practice and improve.”

(A127)

### Improved attitude towards future training on SI conversations

A few EM providers reflected on the EM Talk training and stated that *“[the training] is extremely applicable to our practice. I would recommend all EM doctors undergo training such as this” (B152)*. Other EM providers referred to the effectiveness of the small group discussion format and the ability to download the VitalTalk app for future reference.

“I had a great time in the small groups practicing difficult conversations. I also was happy to get the app downloaded to keep some very useful tools on hand”.

(A84)

### Improved Practice

The theme of improved practice was referenced by 25 respondents. The majority of these reflected the subtheme of commitment to using acquired skills in clinical practice (n = 20) while a minority of respondents (n = 5) stated that were already utilizing taught skills in clinical practice (Table 5).

### Commitment to using acquired skills in clinical practice

The commitment to using acquired skills in clinical practice was indicated by providers who shared a plan to “*incorporating this style of talking about goals of care with my patients and families” (A63)*. A provider acknowledged the ease of acquiring SI conversation skills and that it might take some time for the skill to become second nature.

“It [the training] was interesting and the tool is easy to follow so it should be easy to incorporate into practice. I suspect it will be more comfortable with time and eventually become second nature”.

(A82)

### Already utilizing taught skills in clinical practice

A few EM providers expressed that, between the training completion and survey completion, they had been in clinical scenarios where they had to use some of the SI conversational skills taught. One provider stated that “I feel better about approaching end-of-life discussions and have had some success in my recent practice” (A69). Also, another provider attributed the success in navigating SI conversations he recently experienced to the training he received.

“The very next day I had a patient/family interaction that I was able to identify and navigate because of the training”

(A104)

## Discussion

We report that across the 33 EDs enrolled in the PRIM-ER study, over 85 percent of the EM providers completed the EM Talk training and we estimate that these trained providers will reach approximately 16,389 seriously ill older adult patients that visit the ED. Also, we report that across the thematic domains of improved knowledge, attitudes, and practice, the EM providers reported that the training improved their SI communication skills, improved their attitude towards engaging qualifying patients in SI conversations, and encouraged their commitment to using these learned skills in clinical practice.

The extensive reach of the EM Talk training is noteworthy and this success is the reflection of the commitment of the departmental leadership of each site and their willingness to integrate the training into the educational curriculum in their departments. Also, we selected EM physician champions that were tasked with disseminating the information about the EM Talk training and facilitating attendance. The selection of appropriate and influential clinical champions is pivotal to the successful engagement and training of providers. Earlier studies have reported that clinical champions are instrumental in the quicker initiation of interventions, assist in overcoming institutional barriers, and can motivate and involve staff in clinical trials.^39,40^

We report that every year, a trained EM provider will reach between five and 115 seriously ill older adult patients depending on the ED volume, patient mix, and geographic setting, among other factors. This wide range of encounter highlights the diversity in the patient population that visit the ED, and the need for each ED to conduct a needs assessment, create ED-specific standard operating procedures in engaging qualifying patients in SI conversations, and provide a conducive environment for SI conversations in their respective EDs.^41–44^ Engaging in SI conversations is never an easy task, and creating an enabling environment within the ED for EM providers to engage in such conversations may lighten the burden of delivering bad news and engaging patients in end-of-life goal discussions. Earlier studies have reported that some of the barriers EM providers and emergency nurses face in engaging qualifying patients in SI conversations include lack of privacy, limited patient engagement time, and the fast-paced ED work culture.^9,29,45,46^

EM Talk was designed to provide SI communication skills training to EM providers. Consistent with the goal of the intervention, the EM providers reported that they acquired tips and tricks in navigating SI conversations, are willing to engage qualifying patients in SI conversations, and have the intent to incorporate these learned skills in clinical practice. The observed harmony between the expected goal and self-reported outcome may be explained by the evidenced-based pedagogical technique employed in delivering the EM Talk training. VitalTalk – the parent program from which EM Talk emerged has consistently prioritized role play and small group learning sessions as a bedrock of successful training sessions.^11,12^ Similarly, other authors that taught GeriTalk – another derivative of the VitalTalk, reported that Geriatric and Palliative Medicine fellows had high levels of satisfaction after they underwent the training.^16,20^ Similarly, Berg et al.^47^ reported that Oncology fellows self-reported significant improvement in SI communication skills after undergoing OncoTalk training.

This study has its limitations. Although a large proportion of full-time EM providers completed the training, it is unlikely that all EM providers will embrace and utilize the SI communication skill in their practice. The estimated average of seriously ill patients that would be reached yearly, therefore, represents the best-case scenario. On the other hand, EM providers may learn from one another and the training and knowledge may even spread to those who are not formally trained– i.e., adoption of behavior due to peer influence. There is a possibility that attitude and practice towards engaging qualifying patients in SI conversations will differ by age, race/ethnicity, religious affiliation, and years of practice. Third, differences in the pedagogical styles of the different facilitators may positively or negatively influence the knowledge, attitude, and practice of EM providers toward engaging qualifying patients in SI conversations. Also, the EM Talk started as an in-person training program but was transitioned into an online training session. It is unknown to what extent this change in pedagogy affects the reach and effectiveness of the intervention. Despite these limitations, this study is one of the few that assessed the effectiveness of EM Talk training across the domains of knowledge, attitude, and practice. Also, this is one of the few studies that used the RE-AIM framework to assess the reach and effectiveness of a provider-focused intervention. Furthermore, this study is strengthened by its spread across over 30 EDs and its large sample size.

## Conclusion

The EM Talk training reached a substantial proportion of EM providers working in the 33 EDs enrolled in the PRIM-ER study. The effectiveness of the EM Talk training was reflected across the thematic domains of improved knowledge, attitude, and practice evidenced by EM providers’ self-reported acquisition of tips and tricks in navigating SI skills, willingness to engage qualifying patients in SI conversations, and intent to incorporate the learned skills into clinical practice. Future studies may assess to what extent learned communication skills translate into the proportion of qualifying seriously ill older adults with documented end-of-life goals and the proportion successfully transitioned to comfort care.

## Figures and Tables

**Table 1:** Statistical Definitions of the Reach of the Primary Palliative Care for Emergency Medicine (PRIM-ER) study

Term	Statistical Definitions
Seriously ill (SI) patient	*Meets the following criteria*: 66 years and older Visited one of the 33 EDs at least once Has a GAGNE > 6
Proportion of EM providers trained	*Total Number of EM Providers that Completed EM Talk Training* *Total Number of EM Providers in Participating ED*
Yearly average of SI patient visits	*Total Number of SI patients that had unique ED visits between* 2018 *and* 2020 3 (*the number of years*)
Estimated number of SI patients reached	*Yearly average of unique SI patients that visited the* 33 EDs* Proportion of EM provider strained
SI patient: EM provider ratio	*Estimated number of SI patients reached* *Proportion of EM provider strained*

**Table 2:** Codebook

Theme	Description	Inclusion	Exclusion
Improved Knowledge	Improved or augmented comprehension, understanding, or command of hospice and palliative care practice	Include any item that refers, explicitly or implicitly, to an individual’s improved knowledge in hospice and palliative care practice, with or without specific details	Exclude if the statement refers to the course and does not reflect on individual or group improved knowledge. For implicit meaning: Exclude “close code but not exact” and “no, code is different” after applying the synonym rule
Improved Attitude	A positive feeling or disposition towards hospice and palliative care practice	Include any item that refers, explicitly or implicitly, to an individual’s improved attitude in engaging in hospice and palliative care, with or without specifics	Exclude if the statement refers to the course and does not reflect on individual or group improved attitude. For implicit coding: Exclude “close code but not exact” and “no, code is different” after applying the synonym rule
Improved Practice	Improved day-to-day activities and expertise in engaging hospice and palliative care discussion	Include any item that refers, explicitly or implicitly, to an individual’s improved practice or acquisition of skills in hospice and palliative care, with or without specific details	Exclude if the statement refers to the course and does not reflect on individual or group improved clinical practice or skill acquisition. For implicit coding: Exclude “close code but not exact” and “no, code is different” after applying the synonym rule

Synonym rule: For items that have implicit meanings, a synonym of the anchor word or phrase is applied and the sentence is re-assessed and categorized as either “yes, code is exact”, “no, code is different”, or “close code but not exact”.

**Table 3:** Reach of the EM Talk Training Across the Participating Emergency Departments

Hospital Name	Number of EM Providers Trained	Total Number of EM Providers	Percent Trained (%)	Average Annual Index Visits of Qualifying SI Patients	Potential Number of SI Patients Reached/Year	Yearly SI Patient: EM Provider ratio
Allegheny General Hospital	16	16	100.0	330	330	20.6
Baystate	33	35	94.3	915	863	26.2
Baystate Franklin	9	9	100.0	182	182	20.2
Beaumont Royal Oak	10	11	90.9	1265	1150	115.0
Beaumont Troy	15	18	83.3	1091	909	60.6
Bellevue Hospital Center	15	18	83.3	97	81	5.4
Brigham and Women’s Hospital	19	21	90.5	1054	954	50.2
Brigham and Women’s Faulkner	71	78	91.0	309	281	4.0
Christiana Hospital	33	44	75.0	892	669	20.3
Henry Ford Hospital	45	50	90.0	321	289	6.4
Henry Ford West Bloomfield	22	22	100.0	457	457	20.8
Henry Ford Fairlane	29	32	90.6	144	130	4.5
Hospital of the Univ of Penn	33	36	91.7	683	626	19.0
Mayo Austin Albert Lea	12	16	75.0	262	197	16.4
Mayo Mankato	17	22	77.3	367	284	16.7
Mayo St Mary	51	53	96.2	1162	1118	21.9
MD Anderson	21	26	80.8	1521	1229	58.5
Mount Sinai Beth Israel	16	19	84.2	281	237	14.8
Mount Sinai Hospital	47	48	97.9	722	707	15.0
Mount Sinai West	36	37	97.3	467	454	12.6
NYU Brooklyn	25	31	80.6	715	576	23.0
NYU Long Island	40	45	88.9	1100	978	24.5
Ochsner Medical Center	30	34	88.2	468	413	13.8
OSU Wexner Medical Center	49	78	62.8	800	502	10.2
Penn Presbyterian	15	20	75.0	305	229	15.3
Pennsylvania Hospital	10	13	76.9	280	215	21.5
UCSF Medical Center	15	18	83.3	623	519	34.6
UF Health Shands Hospital	23	31	74.2	215	160	7.0
UF Kanapaha	9	10	90.0	49	44	4.9
UF Springhill	11	13	84.6	141	119	10.8
University of Utah Hospital	35	39	89.7	490	440	12.6
Yale New Haven Hospital	33	42	78.6	1073	843	25.5
Zuckerberg SF General	34	44	77.3	264	204	6.0
**Total**	**879**	**1029**	**85.4**	**19045**	**16389**	**18.6**

Average SI Patients Qualifying Index Visits: Number of patients 66 years and older with an index ED visits who had a GAGNE index of six or higher. The average is calculated by dividing 2018, 2019, and 2020 counts by 3. Estimated SI Patients Reached/Year = Percent Trained * SI Patients Qualifying Index Visits; Yearly SI Patient: EM Provider ratio = Estimated SI Patients Reached/Number of EM Providers Trained; OSU; Ohio State Univerisy: UF: University of Florida; UCSF: University of San Francisco; NYU: New York University; Univ of Penn: University of Pennsylvania

**Table 4:** Content Coding

Theme and Subthemes*	Code Counts
**Improved Knowledge (N = 60)** **	
Acquired SI communication skills	44
Acquired general useful knowledge	14
Acquired empathy skills	3
**Improved Attitude (N = 45)**	
Attitude towards engaging in SI conversations	30
Attitude towards improving patient care	10
Attitude towards receiving future training in SI conversations	5
**Improved Practice (N = 25)**	
Commitment to using acquired skills in clinical practice	20
Already utilizing taught skills in clinical practice	5

* Themes in bold;

** Multiple coding categories across subthemes account for the sum exceeding the total

**Table 5:** Apriori themes, emerged subthemes and the associated meaning units

Theme	Subtheme	Code label	Meaning Units
**Improved Knowledge**	Acquired SI communication skills	Acquired talking techniques in framing discussions	“Learned some techniques to talk to the family of palliative patients”
	Acquired useful general knowledge	Good learning experience	“This was a pretty good learning experience for me”
	Acquired empathy skills	Acquired empathetic skills	“…Learned a lot about empathetic skills that I can use in daily practice”
**Improved Attitude**	Attitude towards engaging in SI conversations	Comfortable and at ease	“.helped me become more comfortable and at ease with end-of-life conversations”
	Attitude towards improving patient care	I see the value	“I see the value it brings to patients and their families”
	Attitude towards receiving future training in SI conversations	Extremely applicable	“…it is extremely applicable to our practice. I would recommend all EM doctors undergo training such as this”
**Improved Practice**	Commitment to using acquired skills in clinical practice	I will incorporate skills into practice	“I look forward to incorporating this style of talking about goals of care with my patients and families”
	Already utilizing taught skills in clinical practice	I already used learned skill	“The very next day I had a patient/family interaction that I was able to identify and navigate because of the training…”

## Data Availability

The datasets used and/or analysed during the current study are available from the corresponding author on reasonable request.

## Abbreviations

RE-AIM: Reach, Effectiveness, Adoption, Implementation, and Maintenance
EM: Emergency Medicine
SI: Serious Illness
ED: Emergency Department
SRQR: Standards for Reporting Qualitative Research
PRIM-ER: Primary Palliative Care for Emergency Medicine
ELNEC: End-of-Life Nursing Education Consortium
CME: Continuing Medical Education
CMS: Centers for Medicare & Medicaid Services
KAP: Knowledge, Attitude, and Practice
MDs: Medical Doctor
